# Sugar, Invertase Enzyme Activities and Invertase Gene Expression in Different Developmental Stages of Strawberry Fruits

**DOI:** 10.3390/plants11040509

**Published:** 2022-02-14

**Authors:** Hayat Topcu, Ipek Degirmenci, Duygu Ayvaz Sonmez, Aibibula Paizila, Harun Karci, Salih Kafkas, Ebru Kafkas, Sezai Ercisli, Aishah Alatawi

**Affiliations:** 1 Department of Agricultural Biotechnology, Faculty of Agriculture, Namik Kemal University, Tekirdag 59030, Turkey; 2 Department of Horticulture, Faculty of Agriculture, Cukurova University, Adana 01330, Turkey; ipek-016@hotmail.com (I.D.); hkarci@cu.edu.tr (H.K.); skafkas@cu.edu.tr (S.K.); ebruyasakafkas@gmail.com (E.K.); 3 Yaltir Agricultural Products Inc., Sarihuglar, Algida 51-1, Adana 01355, Turkey; duygu.ayvaz@gmail.com; 4 Department of Laboratory Technologies, Imamoglu Vocational School, Cukurova University, Adana 01330, Turkey; apaizila@cu.edu.tr; 5 Department of Horticulture, Faculty of Agriculture, Ataturk University, Erzurum 25240, Turkey; 6 Department of Biology, Faculty of Science, University of Tabuk, Tabuk 71421, Saudi Arabia; amm.alatawi@ut.edu.sa

**Keywords:** ‘Rubygem’ and ‘Fortuna’, sweetness, sugar accumulation, HPLC analysis, invertase, qRT-PCR

## Abstract

The cultivated strawberry (*Fragaria* × *ananassa*) is octoploid (2n = 8x = 56) and has been the focused fruit species of which an increasing number of molecular and genetic research has been conducted in recent years. The aim of this study is to identify the relationships between sucrose metabolism, invertase enzyme activity and gene expression in four different fruit development periods (red, pink, green and white) of two commercially important strawberry varieties ‘Rubygem’ and ‘Fortuna’. The metabolite profiles (glucose, fructose, sucrose and total sugar content) of two varieties were discovered to be extremely similar. The highest amount of total sugar was found in red fruits, while the lowest was obtained from green fruits. Invertase represents one of the key enzymes in sucrose metabolism. The lowest invertase activity was obtained from the green fruits in ‘Rubygem’ and ‘Fortuna’ during four developmental periods. In these varieties, the amount of sucrose was found to be close to glucose and fructose and the lowest amount was detected in green period, while invertase activity was relatively high during red and pink periods and invertase gene expression was determined at high levels in both primers (St-4 and St-6) in the green period. The results of the study indicated that sugar content and invertase activity were positively correlated while enzyme activity and gene expression were negatively correlated.

## 1. Introduction

The cultivated strawberry (*Fragaria* × *ananassa*) emerged from the interspecific hybridization of two wild octoploid species. It is one of the most widely consumed berries and economically important as it is recognized as a powerful source of natural antioxidants [1,2,3,4]. Today, strawberries are grown in all continents with more than 20 species and a great number of varieties [4,5]. Strawberries are accepted as a model system for non-climatic fruit metabolism and transcript profiling studies [6] and proposed as a model system for berries due to its short growth cycle and small size [7]. Thus, strawberries have become increasingly valuable as it attracts an increasing number of scientific research and bears great economical potential.

Yield and quality have become a major concern for many strawberry breeding programs in recent years. Strawberry yields can be increased by using new varieties and proper cultural practices. However, fruit quality represents a combination of a wide range of traits such as aroma, sweetness, fruit shape, fruit color, texture and firmness [8]. Among them, sweetness signifies one of the most desirable traits in commercially grown strawberry varieties especially for fresh markets. Sweetness is largely defined by the relative amounts of sugar and organic acids and is often evaluated by trained professional food tasters by comparing them to known commercial varieties. Organoleptic evaluation is greatly influenced by the relative and total sugar and acid amounts in mature fruit [9]. 

Strawberries accumulate sugars and organic acids during fruit maturation [10,11]. In most plants, sucrose is the primary carbohydrate obtained from photosynthetic activity. In strawberries, more than 95% of the translocated sugars are in the form of sucrose [12]. Sucrose content in the fruit is identified by the level of sucrose accumulation and sucrose degradation. The accumulation of sucrose is affected by its carbohydrate accumulating power, biosynthetic and respiratory processes [13]. The rate of carbohydrate degradation is influenced by the proportion at which sucrose is cast out from the cytosol by enzymatic breakdown or transport to vacuole for storage [14]. Depending on the type of cells that distribute carbohydrates, sucrose is decomposed by either sucrose synthase or invertase [15]. Invertase (EC 3.2.7.26) is an enzyme that catalyzes the hydrolysis reaction of sucrose to form glucose and fructose and is extremely important in the regulation of sucrose movement, storage and utilization in many plants. Invertase activity differs across different fruit developing stages. Lee et al. [16] studied the changes in invertase enzyme activity in strawberries during four different fruit development stages and significant changes were detected during or before ripening. Invertase activation increased in the pre-ripening period of the fruit. However, sugars significantly decreased before the ripening phase. Sugar content and enzyme activities at different developmental stages during the fruiting phase bear a great effect on the growth and development of strawberries. During the early growth stage, invertase activity was relatively high but decreased with the accumulation of sucrose. Similar accumulation stages were also reported in other plant species, such as in sugar beet [17], sweet melon [18], mango [19], mandarin [20] and red bayberry fruits [21]. 

Molecular studies often target specific biosynthetic pathways that are effective along the maturation [22]. Studies with gene expression have shown that the activation of enzymes of plants at different stages of development roles bear an extremely major act in the complex molecular mechanism of the plant [23,24]. Although there are some studies which were conducted related to sugar content [25] and enzyme activity [8] in strawberries, there remains no research investigating the relationship between gene expression, enzyme activity and sugar content in strawberries.

In this research, we aimed to determine the correlation among gene expression differences, invertase activity and amounts of sucrose, fructose and glucose during four different development stage of two strawberry varieties, ‘Rubygem’ and ‘Fortuna’. ‘Rubygem’ bears medium to large fruits, conical in shape, large, sweet, highly flavored and red in color. It is sensitive to powdery mildew and resistant to fusarium. It is known for its earliness and its extremely popular taste. It is far easier to transport than the other strawberry varieties. It is a preferred variety for domestic markets and exports [26]. ‘Fortuna’ contains an earliness trait in the regions where production is undertaken during the winter–spring periods. It is distinguished from the other varieties with its highly large, attractive and uniformly shaped marketable fruits produced in the early season. Fruits are large and conical shaped, and fruit surface is medium red. It is one of the most important exported varieties in Turkey due to its high fruit quality [27]. 

This study aims to contribute to the existing data on qualitative parameters such as sugar content by determining the sugar content and invertase activity of two popular commercial strawberry varieties, furthermore the relationship between sugar content, enzyme activity and gene expression were also investigated.

## 2. Material and Methods

### 2.1. Plant Materials 

**Figure 1:**
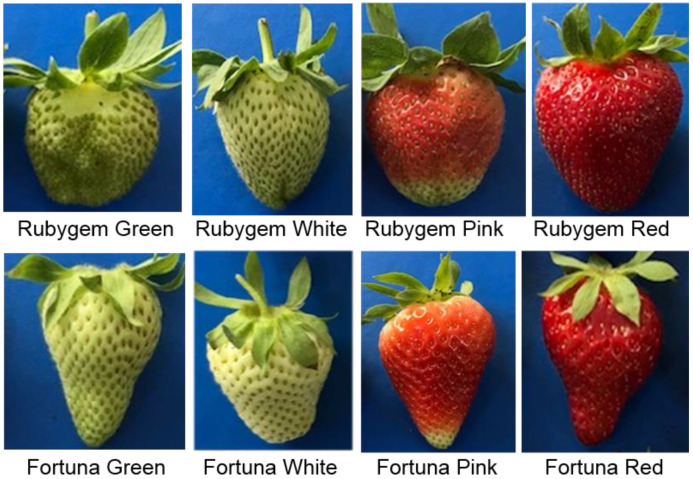
Different fruit developmental stages of two strawberry varieties.

Strawberry varieties ‘Rubygem’ [28] and Florida ‘Fortuna’ [29] (*Fragaria* × *ananassa* Duch.) were harvested from Yaltır Company Research fields located in Adana province of Turkey. At least 10 pieces of fruits were obtained from each variety. The trial design was randomly generated. The samples were selected and grouped after the damaged and diseased strawberries were eliminated. Strawberry fruit samples were categorized into four different developmental stages based on fruit characteristics such as fruit size, color and fruit maturity. As shown in Figure 1, these stages were as follows: green growth stage (container distinguishable from seeds), white (immature yet, achenes are no longer tightly packed), pink (immature yet, but the fruits have taken their full shape and half the color of the fruit is red), red (mature and the fruits have taken their full shape and the fruit color is completely red) [30]. Fruit samples were collected in triplicates during all four different developmental stages from both varieties. Immediately after the berries were harvested, they were submerged into liquid nitrogen at −196 °C and transported to the laboratory at Çukurova University for biochemical and gene expression analyses.

### 2.2. Biochemical Analysis

#### 2.2.1. Characterization of Sugars by HPLC-RID-UV

Changes in glucose, fructose, sucrose and total sugar content in the juice obtained from the harvested strawberries were determined according to the method developed by Akšić et al. [31] Lyophilized strawberry fruit samples were powdered using a mortar and pestle. Then, they were weighed to 0.1 g. A total of 5 mL of ultrapure water (Millipore Corp., Bedford, MA, USA) was added to each sample. The reaction mixture was placed in an ultrasonic bath and sonicated at 80 °C for 15 min, and then centrifuged at 5500 rpm for 15 min and filtered prior to HPLC analysis. (Whatman nylon syringe filters, 0.45 µm, 13 mm, diameter). The high-performance liquid chromatographic apparatus (Shimadzu LC 20A vp, Kyoto, Japan) consisted of an in-line degasser, pump and controller coupled to a refractive index detector (Shimadzu RID 20A vp) equipped with an automatic injector (20 µL injection volume) interfaced to a PC running Class VP chromatography manager software (Shimadzu, Japan).

Separations were performed on a 300 mm × 7.8 mm i.d., 5 µm, reverse-phase Ultrasphere Coregel-87 C analytical column (Transgenomic, Omaha, NE, USA) operating at 70 °C with a flow rate of 0.6 mL min^−1^. Elution was isocratic ultrapure water. Individual sugars were calculated based on their standards and expressed in mg/100 g of dry weight (DW).

#### 2.2.2. Invertase Extraction and Activity Assay 

Invertase (EC 3.2.7.26) was obtained at 4 °C using 50 mM sodium phosphate buffer, pH 7.4 (2 mL per 500 mg fruit) containing 1 mM 3-mercaptoethanol (disulfide bridge reducer). The strawberry fruit samples were ground with the aid of a mortar and pestle and centrifuged at 15,000× *g* for 30 min (with modifications by Morkunas et al. [32] according to Copeland, 1990). The resulting homogenate was centrifuged at 12,000× *g* for 20 min at 4 °C. The pellet formed after centrifugation was discarded and the resulting supernatant was obtained for invertase and protein content measurements. 

Acid and alkaline invertase activity was determined by spectrophotometer utilizing the technique by King et al. [33]. The reagent encloses 120 mL of gist-substance, 480 mL of 100 mM acetate buffer (pH 5 or pH 7.5) and 100 mM sucrose (Table 1). The control reagent includes 120 mL of sucrose-free gist-substance and 480 mL (pH 5 or pH 7.5) acetate tampon as test. All samples were incubated at 30 °C for 1 h. 600 µL of pH 8.3 Tricine-KOH tampon was suffixed to the reagent. The samples were kept at a simmer for 3 min. Afterwards, samples were rapidly cooled and absorbance measurements were performed by spectrophotometer at λ = 560 nm. Enzyme activity was determined as Abs g-1. The activity of invertase was determined in the study of Krishnan and Pueppke [34] with small changes. The experiment was performed by placing 50 mg of fruit juice and 250 µL of sucrose solution in a test tube at 40 °C. The composition was mixed gently during the 30-min reagent time, and then 300 µL of DNS solution was suffixed to the reagent. Next, the samples were kept in boiling water for 10 min and cooled in ice and 4.4 mL of distilled water was added to the reaction. The amount of reduced sugar released was determined spectrophotometrically at 540 nm. A blank reaction without fruit extract was carried out in parallel with the reaction as a control. Invertase activity is defined as the amount of enzyme that produces invert sugar at 1 μmol [35].

Enzyme activity is expressed in terms of total extracted protein. The protein content of the solutions was determined by the AOAC method [36]. Invertase extractions were performed using the DNSA (3,5-Dinitrosalicylic acid) method [37]. 

#### 2.2.3. RNA Isolation and cDNA Synthesis

Total RNA isolation for frozen strawberry samples were performed according to Carvalho et al. [38]. RNA quantity and quality were measured by gel electrophoresis, A260/A280 ratio and Qubit RNA BR assay kit (Invitrogen, Waltham, MA, USA). Then, the RNA samples were adjusted to 100 ng. cDNA synthesis was carried out using the High-Capacity cDNA Reverse Transcription Kit (Applied Biosystems, Thermo Fisher Scientific, Waltham, MA, USA), 5 μL of 100 ng RNA was used for cDNA synthesis.

#### 2.2.4. Real-Time Quantitative (qRT-PCR)

The obtained cDNAs were diluted 5 times and qPCR reactions were performed. The qPCR reactions were performed using RealQ Plus 2× Master Mix Green (Ampliqon, Odense, Denmark) as recommended by the company. The qPCR analyzes were performed using the Roche Lightcycler^®^ 96 (Roche Life Science) instrument. A total of 3 μL of cDNA was used in qRT-PCR reactions. The amplification program consists of 1 cycle of 95 °C for 10 min, followed by 40 cycles of 95 °C for 10 min and 55 °C for 30 s, and 1 cycle of 37 °C for 30 s. Relative expression level was calculated using 2 ^−ΔΔCT^ method and HISTH4 was used as reference gene. The primers used in this study were designed by Universal ProbeFinder version 2.53 (https://lifescience.roche.com/en_tr/brands/universal-probe-library.html (accessed on 12 October 2021)) using the invertase related gene sequences obtained from the study by Shulaev et al. [39]. Primer sequences are listed in Table 2. 

### 2.3. Statistical Analysis

Data are expressed as mean ± standards of two repetitions and analyzed using ANOVA with statistical software of SPSS [40]. All experiments were performed with three biological replicates. The gene expression relative expression level method was used to evaluate the qRT-PCR results. The heatmap was created using gene expression data in R packages gplots and RColorBrewer.

## 3. Results

In this study, fruits of ‘Rubygem’ and ‘Fortuna’ strawberry varieties were evaluated in terms of sugar contents (sucrose, glucose, fructose and total sugar), invertase enzyme activity and invertase expression during four different stages of fruit development, covering the period from growth to ripening stages. ‘Rubygem’ of ‘Fortuna’ and ‘Fortuna’ were sampled starting from the green phase until reaching the red phase that requires 37 and 38 days after anthesis for ‘Rubygem’ and ‘Fortuna’, respectively.

Correlation analysis was performed in five biochemical traits and invertase activity belonging to two strawberry varieties (Table 3). Correlation analysis of soluble sugars such as glucose, fructose and sucrose contents, WSDM (water soluble dry matter = Brix) and invertase activity was performed in two strawberry varieties at four different stages of development. The correlations between all traits were calculated as positive. The highest positive correlation (*p* < 0.001) was detected among fructose and total sugar content values, the lowest positive correlation (*p* < 0.05) was computed between sucrose and invertase activity. 

**Figure 2:**
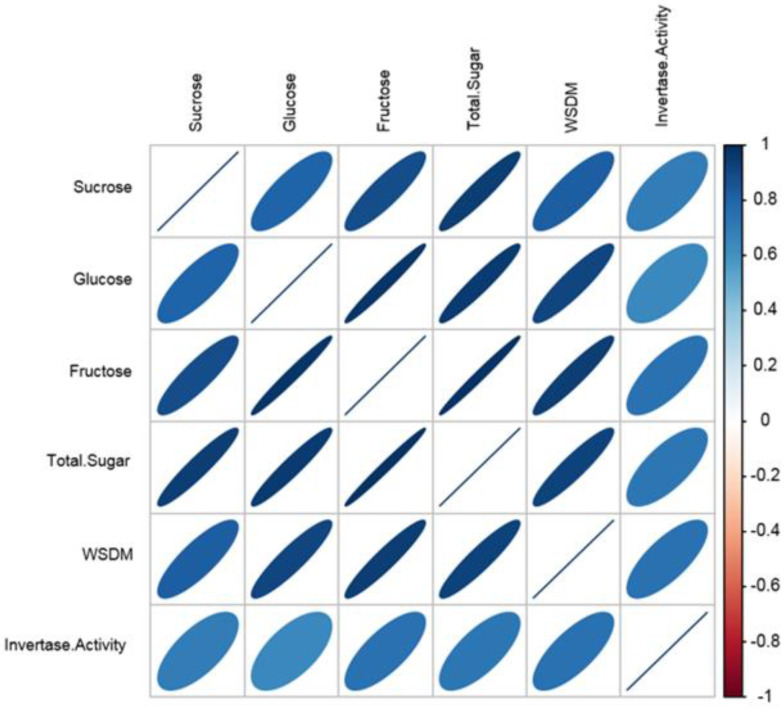
Correlation plot belonging to six different biochemical traits of two strawberry cultivars at four growing stages.

The correlation values of these two features are close to zero, which means that they bear the lowest positive correlation. The correlation graph of six different biochemical characteristics of two strawberry cultivars at four growing stages is given in Figure 2.

### 3.1. Sugar Content

Glucose, fructose, sucrose contents and total soluble sugar content were determined in fruits obtained from four different developmental phases using HPLC in both varieties. Although there was no statistically significant difference between sugars when all sugars were evaluated among all growing seasons in both varieties, total sugar content increased nearly two-fold during berry ripening in both ‘Fortuna’ and ‘Rubygem’ varieties. Total sugar content increased during berry ripening in both ‘Fortuna’ and ‘Rubygem’ varieties, while, glucose content remained nearly consistent throughout the fruit developmental phases. The sucrose content was computed as 7.7 mg/100 g in the green growth stage of ‘Fortuna’ and the content was found as stabile in the white and pink stages, although it reached 21.2 mg/100 g in the mature stage. In ‘Rubygem’, sucrose content was 6.0 mg/100 g in the green growth period, while it was found 16.9 mg/100 g in the pink period. However, it was calculated as stabile in the mature and white stages in ‘Rubygem’. On the other hand, fructose and glucose content of ‘Fortuna’ and ‘Rubygem’ was found to be similar throughout the entire fruit development period. Total sugar content was highest in the mature period in both varieties. Brix values of glucose, sucrose and fructose contents in four different periods in ‘Fortuna’ were computed as 25.0%, 20.0%, 21.6% and 17.0%, respectively. However, the fructose content in soluble sugars was slightly higher than sucrose and glucose contents. The brix values of glucose, sucrose and fructose contents in ‘Rubygem’ were detected as 24.3%, 19.3%, 18.3% and 14.3%, respectively.

### 3.2. Invertase Activity

The specific invertase activity is described as its activity in 1 mg of protein. The specific invertase activity was evaluated in both varieties. The soluble acid invertase activity in ‘Rubygem’ was detected as highest (38 DAFB), while activity in green period was found to be lower than other developmental stages. Invertase activity in early stages of development in both varieties were determined on minimal level and this activity decreased with sucrose reduction in this phase. However, the invertase activity in both varieties demonstrated an increase throughout the process from the green stage to the ripe period.

Soluble acid invertase activity in the green growth stage was discovered lowest 14 days after full bloom (DAFB) in ‘Fortuna’, while it was calculated that the highest activity was in the pink growth phase 30 DAFB. Invertase enzyme activity in the green growth stage in ‘Rubygem’ was determined lowest 13 DAFB and was detected as the highest in mature fruits 37 DAFB. Specific activity increased with sucrose accumulation, however, gene expression activity of invertase decreased with the accumulation of sucrose in both varieties.

### 3.3. Gene Expression

**Figure 3:**
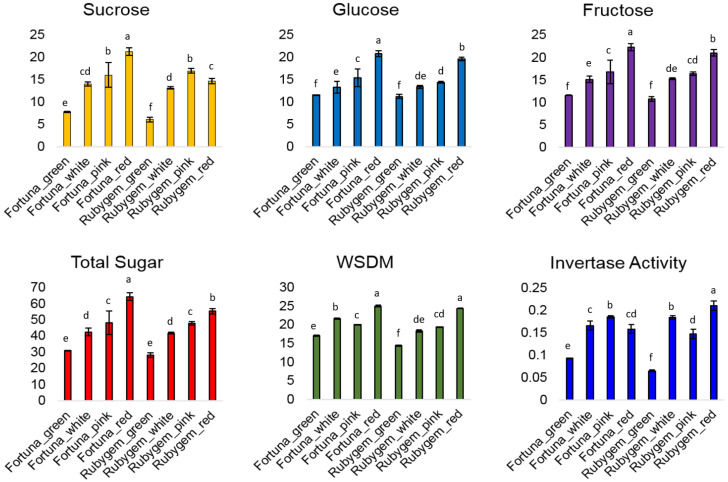
Sugars (mg/100 g fresh weight, mean ± SE of triplicate assays), WSDM (%) and invertase activity (nmol/mg protein, lyophilizer weight) in ‘Rubygem’ and ‘Fortuna’. Different letters on bars indicate statistically significant differences at *p* < 0.05 level.

Invertase gene expression analysis was conducted in ‘Rubygem’ and ‘Fortuna’ strawberry varieties. Samples of four developmental stage of both varieties were tested in seven invertase primers, however, only two primers amplified on these samples. The two primers (St-4 and St-6) showed significant expression differences during four different fruit development stages in two varieties. However, these two primers showed similar expression value in almost all different fruit development stages in both varieties (Figure 3). St-4 and St-6 primers used for the construction of a heatmap was created according to qRT-PCR results of ‘Rubygem’ and ‘Fortuna’ at four different developmental stages using R packages gplots and RColorBrewer. According to the expression results obtained from four different periods of both varieties, invertase gene expression was determined at a high level in both primers in the green period, while no significant difference was observed between the expression values in other periods.

**Figure 4:**
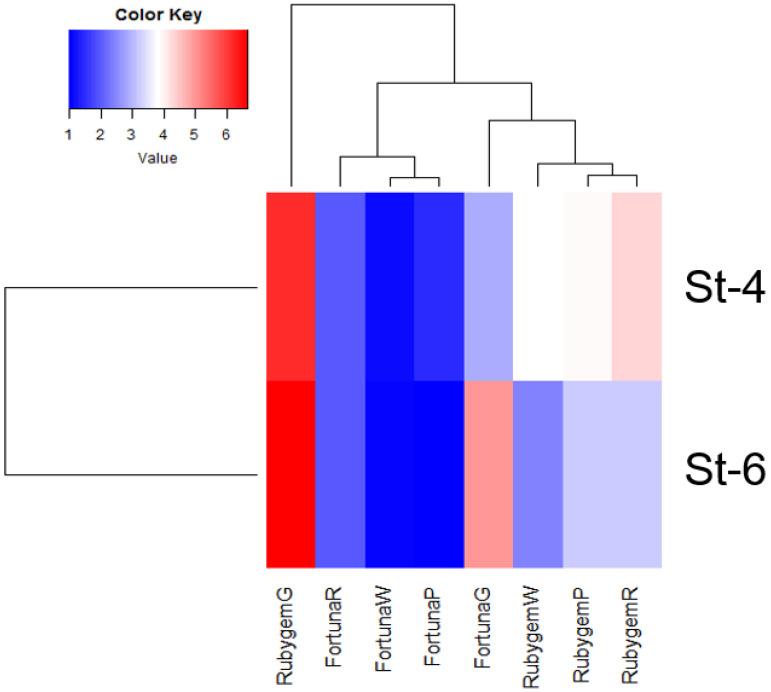
The heatmap of expressed genes related to invertase activity in four different fruit development stages in two strawberry varieties.

The activity of the INV gene in both varieties was determined as highest in the green period, gene activity was observed as lowest in the white period. Fortuna’s gene expression level was discovered as lower than Rubygem’s; however, the INV gene expression level was found to be similar in both varieties. In addition, a negative correlation was observed between INV gene expression level and INV activity and sugar content in fruits at four different stages of development (Figure 4).

## 4. Discussion

In this study, the carbohydrate metabolism, invertase activity and expression profile of the invertase gene of two commercial varieties (‘Rubygem’ and ‘Fortuna’) were evaluated in four different fruit development stages. Moreover, similar research methods were recommended by other researchers since strawberry fruits have different biochemical processes during four different fruit developmental stages [8,35].

### 4.1. Sugar Content and Invertase Activity

Sugar content represents one of the most important quality characteristics in strawberries which can directly affect consumer preference [34]. In ‘Fortuna’ varieties glucose, fructose and sucrose content did not show a significant difference in the fruit ripening (red) stage, while glucose and sucrose were the main sugars in the ripening stage of the variety ‘Rubygem’. Lee et al. [16] stated that sucrose is the main soluble sugar in high sugar-content strawberry varieties, while fructose is the main soluble sugar in low sugar-content varieties. However, Shanmugam et al. [40] reported that fructose and glucose are the main soluble sugars in some strawberry varieties. These difference of soluble sugar content of varieties can be affected by internal factors, such as genotypic differences and also by external factors such as, environmental conditions and agriculture processes [41]. The average total sugar content of strawberries is around 56 ± 0.2 mg/g fresh weight (FW) [8]. In this study, the total sugar content of ‘Fortuna’ and ‘Rubygem’ were 64 ± 0.2 mg/g and 55± 0.2, respectively, which is around the average. However, fructose is 2.3 times sweeter than glucose, and relatively the high fructose level of our two varieties can render them sweeter than average varieties.

Studying the changes in sugar content and enzyme activities during different fruit development stages is extremely important for understanding the sugar metabolism of fruits. However, there is a paucity of similar studies in the literature, especially regarding strawberries. Total soluble sugar and all soluble sugar contents increased nearly two-fold from the green to red stage in ‘Fortuna’ and ‘Rubygem’ varieties in this study. Basson et al. [8] also detected the 1.5-fold change in total soluble sugar between the green stage and red stage in ‘Ventana’ and ‘Festival’ varieties. Moreover, Kafkas et al. [25] found that fructose, glucose, sucrose and total sugar contents increased linearly as the fruits matured. Tian et al. [42] additionally reported an increase in sucrose, glucose and fructose content during fruit development in both strawberries and grapes. All those results are in accordance with our results. Additionally, they detected the highest levels of glucose, fructose and sucrose in red fruits. In our study, invertase activity was detected during all four growing stages in both varieties. Basson et al. [8] also detected the invertase activity during all development stages and this invertase activity can contribute to the sink stress of the berry by breaking down the sucrose. However, the highest invertase activity was observed in pink and white stages in Festival and Ventana, respectively. Ranwala et al. [43] reported an increasing invertase activity during fruit ripening. Li et al. [44] found that invertase activity and sugar content had a positive correlation. Our results are in accordance with the results of Ranwala et al. [43] and Li et al. [44] in which the invertase activity increases with the fruit development process in strawberries.

### 4.2. Gene Expression

Although there are several studies on commercially important strawberry varieties, most of those studies solely focused on carbohydrate content and activation of enzymes involved in carbohydrate metabolism in strawberries [16,40,41]. In addition to these, the expression profile of the INV gene, an enzyme that bears an important role in carbohydrate metabolism and breaks down sucrose into glucose and fructose, was also examined in this study. 

In this study, we investigated the relationship among invertase enzyme activities, invertase gene expression and sugar content in different developmental stages of fruits in two strawberry varieties. According to the results of invertase gene expression, we determined that invertase gene is most active during the green period in both varieties. 

It was determined that sucrose exhibited the greatest difference between these two varieties. The increase in the amount of fructose and glucose due to the expression of the INV gene, which is involved in sugar metabolism, and the decrease in the expression of the INV gene in parallel with the ripening of the fruits, indicates that this gene is related to ripening in these fruits. Lee et al. [16] determined that the difference between glucose, fructose, and sucrose, significantly affects the sweetness of strawberry fruits, moreover, sucrose is responsible for the biggest difference between the two strawberry varieties. They suggested that the *FacwINV2-1* gene, which is highly expressed in varieties with a high sugar content may play an important role in controlling sugar accumulation in strawberry fruits. Researchers with del Olmo et al. [45] studied the gene families involved in sucrose metabolism during three different maturation stages, nearly red (AR), full red (FR), and dark red (DR) of ‘Mara des Bois’ strawberries (*Fragaria vesca*) variety. They claimed that sucrose accumulation in fully ripe strawberries was associated with the significant decrease in FvVIN2 (vacuolar invertase) expression. In this study we also found that while sugar content increased in ripe fruits, invertase activity decreased.

## 5. Conclusions

In conclusion, sugar content, invertase enzyme activity and invertase gene expression in different strawberry fruit development stages were investigated in this study. As a result of this study, the sugar contents of different strawberry varieties were found to vary at different growth stages and in parallel, the invertase enzyme activity also changes according to these developmental stages. The taste and aroma of strawberries are directly affected by sugar metabolism. Thus, determination of the content of carbohydrates during the ripening period of strawberries, expression and activity of enzymes such as invertase that controls sugar metabolism, will aid in the development of new varieties for research and commercial purposes. The results of this study will shed light on biochemical, enzyme and genetic studies not only in strawberries but also in the berry species.

## Figures and Tables

**Table 1:** Enzyme activity assay.

Enzyme	Assay Components	Substrate	Reference
Invertase	120 mL of extract, 480 mL of 100 mM acetate buffer pH 5 or pH 7.5 and 100 mM sucrose	Sucrose	[31,32]

**Table 2:** Primers, primer sequences and annealing temperatures used for gene expression.

Gene ID	Gene	Primers	Forward Primer 5′ to 3′ Reverse Primer 5′ to 3′	Annealing Temperature (°C)
LOC105353049	neutral alkaline invertase 3, chloroplastic-like [*Fragaria vesca* (wild strawberry)]	St-1	CAAGAAGGAAATCGAAGCACAGGCCCAGGAHCATGATTA	55
LOC101304591	alkaline neutral invertase A, mitochondrial [*Fragaria vesca* (wild strawberry)]	St-2	AGGGAGTTTTCGGACATTGAAATACGTCACCACCGACTCC	55
LOC101314895	probable alkaline neutral invertase F [*Fragaria vesca* (wild strawberry)]	St-3	CTAAGCGCATCGCAGCTCGCTGCTTAAAATCAAGCCAGTAG	55
LOC101296189	alkaline neutral invertase A, mitochondrial [*Fragaria vesca* (wild strawberry)]	St-4	GAATTGGCAGAAAAGGCAGTTCAGGCCAATGATCTATAGAAAGC	55
LOC101296831	probable alkaline neutral invertase D [*Fragaria vesca* (wild strawberry)]	St-5	GGTTTTTGTGCGTGACTTTGTCAGGCTCACCATTCATCAG	55
LOC101301732	probable alkaline neutral invertase B [*Fragaria vesca* (wild strawberry)]	St-6	AGTTGCTCCGGTTGATTCTGGATTTTGTGTATGCGCGAAG	55
LOC101292085	alkaline neutral invertase E, chloroplastic [*Fragaria vesca* (wild strawberry)]	St-7	TTGGAGCACGTGAAATGCTTTAAGCGCTTGCATGAGG	55

**Table 3:** Six different biochemical traits belonging to two strawberry cultivars using Pearson correlation test model in R packages corrplot * *p* < 0.05, ** *p* < 0.01, *** *p* < 0.001.

Traits	Sucrose	Glucose	Fructose	Total Sugar	WSDM	Invertase Activity
Sucrose	1	0.8 *	0.88 **	0.94 ***	0.83 *	0.69 *
Glucose	0.8 *	1	0.98 ***	0.95 ***	0.91 **	0.64 *
Fructose	0.88 **	0.98 ***	1	0.99 ***	0.94 ***	0.75 *
Total Sugar	0.94 ***	0.95 ***	0.99 ***	1	0.92 ***	0.73 *
WSDM	0.83 *	0.91 **	0.94 ***	0.92 ***	1	0.75 *
Invertase Activity	0.69 *	0.64 *	0.75 *	0.73 *	0.75 *	1

## Data Availability

All new research data were presented in this contribution.
